# Novel Promising Estrogenic Receptor Modulators: Cytotoxic and Estrogenic Activity of Benzanilides and Dithiobenzanilides

**DOI:** 10.1371/journal.pone.0145615

**Published:** 2016-01-05

**Authors:** Malgorzata Kucinska, Maria-Dolores Giron, Hanna Piotrowska, Natalia Lisiak, Walter H. Granig, Francisco-Javier Lopez-Jaramillo, Rafael Salto, Marek Murias, Thomas Erker

**Affiliations:** 1 Department of Toxicology, Poznan University of Medical Sciences, Poznan, Poland; 2 Department of Biochemistry and Molecular Biology II, School of Pharmacy, University of Granada, Granada, Spain; 3 Department of Clinical Chemistry and Molecular Diagnostics, Poznan University of Medical Sciences, Poznan, Poland; 4 Department of Medicinal Chemistry, University of Vienna, Vienna, Austria; 5 Department of Organic Chemistry, Institute of Biotechnology, School of Sciences, University of Granada, Granada, Spain; Institute of Biomedicine, FINLAND

## Abstract

The cytotoxicity of 27 benzanilides and dithiobenzanilides built on a stilbene scaffold and possessing various functional groups in aromatic rings previously described for their spasmolytic properties was assayed on three human cancer cell lines (A549 –lung adenocarcinoma, MCF-7 estrogen dependent breast adenocarcinoma and MDA-MB-231 estrogen independent breast adenocarcinoma) and 2 non-tumorigenic cell lines (CCD39Lu–lung fibroblasts, MCF-12A - breast epithelial). Three compounds (**6**, **15** and **18**) showed selective antiproliferative activity against estrogen dependent MCF-7 cancer cells and their estrogenic activity was further confirmed in MCF-7 transfected with an estrogen receptor reporter plasmid and in HEK239 cells over-expressing the estrogen receptor alpha (ERα). Compound **18** is especially interesting as a potential candidate for therapy since it is highly toxic and selective towards estrogen dependent MCF7 cell lines (IC_50_ = 5.07 μM versus more than 100 μM for MDA-MB-231) and almost innocuous for normal breast cells (IC_50_ = 91.46 μM for MCF-12A). Docking studies have shown that compound **18** interacts with the receptor in the same cavity as estradiol although the extra aromatic ring is involved in additional binding interactions with residue W383. The role of W383 and the extended binding mode were confirmed by site-directed mutagenesis.

## Introduction

Breast cancer is the most common female malignancy and the second leading cause of cancer related deaths [1]. It is commonly accepted that estrogens influence the normal physiological growth, proliferation and differentiation of breast tissues as well as the development and progression of breast malignancy [2,3]. These effects are normally mediated by 17-β estradiol (E_2_) and related compounds upon binding to two members of the nuclear receptor superfamily: the estrogen receptor (ER) ERα and ERβ [4]. Upon ligand binding, ERs undergo a conformational change which allows chromatin interaction and the regulation of target genes transcription [5].

There are chemical compounds that, albeit binding to the ERs, provoke effects ranging from estrogenic to anti-estrogenic response. Based on this knowledge, selective estrogen receptor modulators (SERMs) were introduced in breast cancer prevention and therapy [6]. SERMs possess different levels of estrogenic agonist or antagonist activity in their target tissues (breast, uterus, bone) [6,7]. Furthermore, SERMs have been studied and received approval for several medical indications (e.g. breast metastatic cancer in women and men, dyspareunia, osteoporosis, vasomotor symptoms associated with menopause) [6,8,9].

Despite most SERMs were shown to decrease the risk of breast cancer, side effects may be relevant [10]. The most widely used SERMs in positive estrogen receptor breast cancer is Tamoxifen. However, its use has been associated with a higher risk for endometrial cancer after long-term treatment [11]. The incidence of uterine cancer in women treated with Raloxifene, another member of SERMs approved for breast cancer chemoprevention, is significantly lower when compared to Tamoxifen treated population, but it may induce thrombosis that may result in ischemic heart disease and fatal stroke in postmenopausal women [12,13]. Despite of the fact that several new SERMs like e.g. Arzoxifene were designed, tested and often mentioned as a promising agents in safe breast cancer prevention, their side effects like coronary events, strokes and bone fractures are still not fully eliminated [14]; therefore Tamoxifen, Raloxifen and aromatase inhibitors are still in use [15]. Additionally, endocrine therapy of ER positive cancers is not always successful since resistance may develop during therapy [16]. In this context, there is still a need for new and more selective and effective SERMs in ER positive cancer treatment and devoid of significant side effects.

In this article, several benzanilide derivatives previously designed and described as spasmolytic agents were tested for their cytotoxic and antiproliferative activity. These compounds were assayed in the A549 lung adenocarcinoma cell line, the lung fibroblasts CCD39Lu, estrogen dependent (MCF-7) and independent (MDA-MB-231) breast cancer cells as well as in the non-tumorigenic MCF-12A breast cell line. Some compounds showed a strong selective cytotoxicity against the estrogen dependent MCF-7 breast cancer cells and our experiments supports that the activity probably is mediated by their interaction with ERα. Compound **18** is a very promising SERM with a potential future application in chemoprotection. Docking and site-directed mutagenesis experiments suggest that it interacts with the receptor in the same cavity as estradiol but forming extra binding interactions with residue W383.

## Materials and Methods

### Chemicals

The tested compounds were synthesized as described elsewhere [17–19]. All chemicals and cell culture supplements, unless otherwise stated, were obtained from Sigma-Aldrich (St. Louis, Mo, USA), penicillin-streptomycin solution was from Gibco Invitrogen Corp. (Grand Island, NY, USA). Plasmid pEGFP-C1-ERα was generously provided by Professor M. Mancini (Department of Molecular and Cellular Biology, Baylor College of Medicine, Houston, Texas 77030, USA, Addgene plasmid #28230). Plasmid p3xERRE/ERE-luciferase was kindly provided by Professor R. Riggins (Department of Oncology, Georgetown University School of Medicine, Washington, DC, USA, Addgene plasmid #37852).

### Cell lines

The cell lines used in the cytotoxicity study, besides MCF-12A, were purchased from European Collection of Cell Culture (ECACC, Salisbury, UK): HEK293 (human embryonic kidney, ATTC no. CRL-1573), A549 (Human Caucasian lung carcinoma, ECACC no. 86012804), CCD39Lu (Human Caucasian lung fibroblasts, ECACC no. 90110512), MCF-7 (Human Caucasian breast adenocarcinoma, ECACC no. 86012803) and MDA-MB-231 (Human Caucasian breast adenocarcinoma ECACC no. 92020424). Cell lines were cultured in DMEM medium without phenol red and supplemented with 10% (v/v) FBS, 1% penicillin-streptomycin and 1% L-glutamine. MCF-12A (ATTC no. CRL-1573) was purchased from American Type Culture Collection (ATCC Manassas, VA 20110 USA). MCF-12A cells were kept in 1:1 mixture of Dulbecco’s modified Eagle’s medium and Ham’s F12 medium, 20 ng/ml human epidermal growth factor, 100 ng/ml cholera toxin, 0.01 mg/ml bovine insulin, 500 ng/ml hydrocortisone and 5% horse serum. All cell lines were cultured at 37°C, in a humidified atmosphere containing 5% CO_2_.

### MTT assay

Compounds were dissolved in DMSO to the final concentration of 100 μM. A549, CCD39Lu, MCF-7, MDA-MB-231 and MCF-12A cells were seeded in 96 wells plates at density 2x10^4^ cells/well and incubated overnight as described above. Subsequently, cells were treated with tested compounds at concentrations: 100 μM; 50 μM; 25μM; 12.5μM; 6 μM; 3 μM; 1.5 μM. Compound **10**, due to its high cytotoxicity, was additionally assayed at concentration range 1 μM; 0.5 μM; 0.25μM; 0.125μM; 0.06 μM; 0.03 μM; 0.15 μM; DMSO was used as control and the concentration in medium did not exceed 0.1%. Cells were incubated 72 h at 37°C in a humidified atmosphere containing 5% CO_2_ in the cell culture media appropriate for each cell line. Then, the medium was aspirated and 170 μl MTT solution (5mg/ml) was added to each well. Plates were incubated for 2 hours at 37°C, and then plates were centrifuged at 1200 rpm for 3 minutes. The formazan crystals were dissolved in 200 μl DMSO and plates were further agitated on a plate shaker at 300–500 rpm for 10 minutes. Absorbance at 570 nm (with reference wavelenght 650 nm) was measured using a plate reader (Biotek Instruments, Elx-800). Cell viability was calculated as a percentage relative to the control.

### Sulphorodamine B assay

In addition to the MTT assay, cell viability was measured by the Sulphorodamine B assay according to Vichai and Kirtikara [20]. The MCF-7 and MDA-MB-23 cells were seeded and treated as for the MTT assay. After incubation the medium was removed and the cells were fixed with cold (4°C) trichloroacetic acid (final concentration 10%) for 1h. Than plates were washed 4 times with water and stained for 30 min, following by staining with sulforhodamine B (SRB) 0.4% in 1% acetic acid solution at room temperature for 20 min. After staining, the SRB solution was removed and the plates washed 5 times with 1% acetic acid dried. Bound SRB was solubilized with 200 μl 10 mM unbuffered Tris-base solution and plates were shaked for 10 min. The absorbance at 510 nm was measured using a plate reader (Biotek Instruments, Elx-800).

### LDH release assay

Cytotoxicity was evaluated by measuring the cell membrane permeability, using a LDH assay kit purchased from Cayman Chemical (Ann Arbor USA). The LDH assay was performed for MCF-7 and MDA-MB-231 cells (seeded at 2×10^4^ density). As a positive control cell permeabilization with 1%Triton X-100 was used. The assay was performed 72h after start the incubation. Briefly, the plates were centrifuged at 400 ×g for 5 minutes, and 100 μL of supernatant were transferred from each well to the new 96-well plate and analyzed according to the manufacturer’s protocol. 100 μL of reaction mixture, containing lactic acid, NAD^+^, tetrazolium salt and diaphorase, was added to each well. Plates were incubated on an orbital shaker for 30 minutes at room temperature and the absorbance at 490 nm was measured with a plate reader (Biotek Instruments, Elx-800). Results were expressed as a percentage of the total value of the positive control.

### Estrogen Receptor luciferase assays

To obtain a eukaryotic vector expressing only the ER-α nuclear receptor, a construct was designed by cloning a *Kpn*I-*BamH*I fragment from plasmid pEGFP-C1-ERα containing the ERα coding sequence that includes a stop signal into the pEGFP-N3 plasmid (Clontech Laboratories, Palo Alto, CA) under the control of the CMV promoter. This construction was termed pEGFP-N-ERα, and although it encoded both human ERα receptor and GFP protein, only the former is expressed. For mock transfections, pEGFP-N3 plasmid was digested with *Bgl*II and *Not*I and religated after polishing DNA ends to remove GFP coding sequence. Plasmid sequence was confirmed by automatic sequencing. Prior to transfection, cells were seeded in 48 well plates at density of 1.5 x 10^4^ cells/well and incubated for 24 h to reach a cell confluence of 80–90%. HEK293 cells, which do not express ERα receptor, were transfected with a mixture of pEGFP-N-ERα, p3xERRE/ERE-luciferase plus the pRLTKRenilla luciferase control plasmid (50:45:5) using LipofectAMINE 2000 (Invitrogen, Carlsbad, CA, USA), as described by the manufacturer. MCF-7 cells, which endogenously express ERα receptor, were transfected with p3xERRE/ERE-luciferase plus the pRLTK Renilla luciferase control plasmid (95:5). Firefly and Renilla luciferase activities were measured using the Dual Luciferase Assay System (Promega, Madison, WI, USA), following a standard protocol using a Sirius L luminometer (Berthold Technologies, Bad Wildbad, Germany). Data were expressed as relative changes in luciferase activity and normalized to 100%.

### Docking Studies

Ghemical 2.95 [21] was used to generate the 3D coordinates of the ligands and for geometrical minimization by molecular mechanics with the tripos 5.2 forcefield until the gradient energy was lower than 0.001 Kjul/mol. The coordinates of the proteinwere taken from the structure deposited in PDB with the accession code 3td3 [22]. Docking studies were carried out at SwissDock sever [23] in accurate mode and without defining the region of interest (blind docking) but allowing flexibility for the side chains within 5 Å of any atom of the ligand in its reference binding mode. Docking results were sorted by their fullFitness scores, a parameter that accounts for the solvation free energy [24]. Analysis of the results was carried out with the help of UCSF Chimera [25], Swiss PdbViewer [26] and Poseview [27].

### ERα Site directed Mutagenesis

Plasmids for the expression of the ERα W383A and ER-α W383S mutated receptors were constructed by the quick change site directed mutagenesis method [28] using plasmid pEGFP-N-ERα as template. For the mutagenic reaction, forward and reverse mutagenic oligonucleotides were used, being the W383A forward oligonucleotide 5´-CCACCTTCTAGAATGTGCC**GCG**CTCGAGATCC-3´ and the W383S oligonucleotide 5´-CCACCTTCTAGAATGTGCT**AGC**CTAGAGATCC-3´. Indicated underlined are new *Xho*I and *Nhe*I restriction sites generated by silent mutations to allow rapid screening. The bases that codify for the mutated amino acids at position 383 are in bold. Plasmid sequences were confirmed by automatic sequencing.

### Statistical analysis

Results are expressed as mean ± SEM. Differences between mean values of control and problem groups were analyzed by an unpaired t-test and between mean values of multiple groups by one way ANOVA followed by Tukey test as appropriate. *P* < 0.05 was considered statistically significant.

## Results and Discussion

Compounds built on benzanilide scaffold have interesting biological activities, however reports concerning their properties are still very sparse. To date only a few papers describing their antifungal [29] and antimycobacterial [30] properties have been published. Additionally, several recent reports have shown that benzanilide derivatives can act as HIF-1 inhibitors [31], histone deacetylase (HDAC) inhibitors [32], α_1_-adrenoceptor antagonists and steroid 5α-reductase inhibitors [33] as well as quinone reductase 2 inhibitors [34]. Moreover, Calderone and coworkers showed that benzanilide derivatives can act as calcium-activated potassium channels activators, being N-(2-hydroxy-5-chloro)-2-methoxy-5-chloro-benzanilide the most active compound [35]. In previous studies, the anti-spasmolytic activity of ten benzanilide derivatives [17] and twenty thiobenzanilide derivatives [18] has been also evaluated.

It has been shown that thiobenzanilide derivatives can act as anticancer agents towards melanoma cells [36] and is has been reported that the benzamidine moiety bearing N-hydroxy-N'-(3,4,5-trimethoxphenyl)-3,4,5-trimethoxy-benzamidine, possesses antiproliferative activity and dose dependent capability to induce apoptosis against human pancreatic cell lines [37].

**Fig 1:**
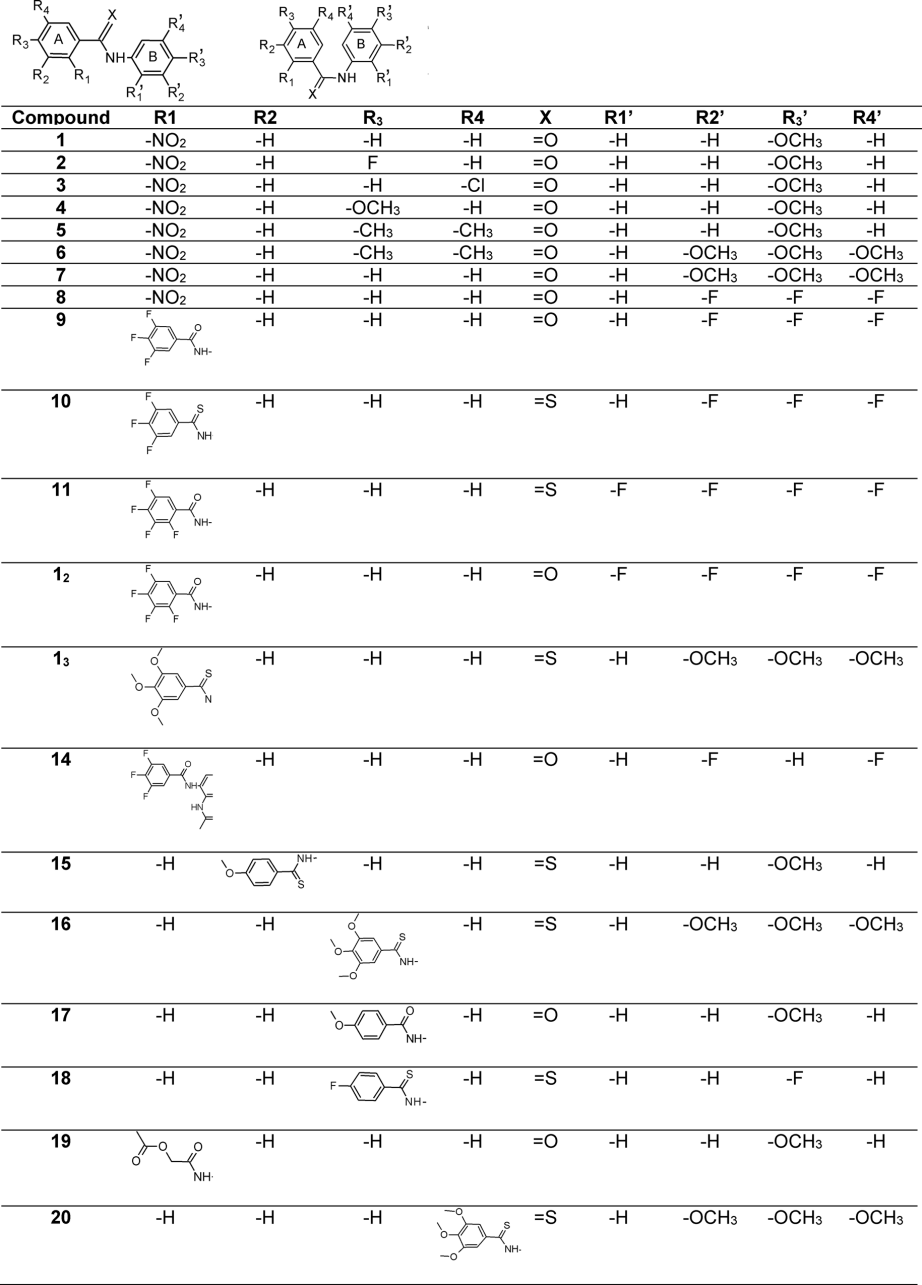
Structures of tested compounds, all compounds possess *trans* conformation except compounds 19 and 20.

In this context, we decided to screen the anticancer activity of a library of benzanilide and thiobenzanilide derivatives shown in **Fig 1** on two lung and three breast cell lines. The lung derived cells were: A549 adenocarcinoma and non-tumorigenic lung fibroblasts CCD39Lu. The breast derived cell lines used were: the estrogen dependent adenocarcinoma MCF-7, the estrogen independent adenocarcinoma MDA-MB-231 [38] and the non-tumorigenic breast epithelial MCF-12A cells. The effects of these compounds on cell viability were measured by a MTT assay as presented in **Table 1.** In general, the activity of the compounds as well as their specificity was dependent on their chemical structure, being compounds **10** and **11** highly active towards all the cell lines assayed. In the case of compounds with equivalent substituents, the nature of X seems to be relevant since those with the thioamide moiety (compounds **10** and **11**) are more cytotoxic than their amide counterparts (compounds **9** and **12**). Moreover, for compounds containing thioamide as a linker, the presence of fluorine atom (at positions R1’/R2’/R3’ or additionally R4’) increased the cytotoxicity (compounds **10**, **11**). A relatively homogenous structural group is represented by compounds **1–8** that share the substituents at positions R1 (-NO_2_), R2 (-H), R1´ (-H), R3´ (–OCH_3_) and +X being oxygen. In this group compounds **2** and **4** showed activity against both lung and breast cancer cell lines, suggesting the importance of the substitution at R3.

**Table 1:** Cytotoxic activity (IC_50_) of tested compounds against selected breast and lung derived cell lines.

	Cell line IC50 [μM]^a^				
	MCF-7	MDA-MB-231	MCF-12A	A549	CCD39Lu
COMPOUND					
**1**	74.87	79.80	>100	92.38	96.32
**2**	38.29	39.67	>100	47.64	45.18
**3**	>100	>100	>100	>100	>100
**4**	28.36	66.99	>100	30.12	48.83
**5**	>100	91.98	>100	>100	>100
**6**	24.65	>100	>100	>100	>100
**7**	>100	>100	>100	93.08	>100
**8**	>100	>100	>100	>100	>100
**9**	>100	>100	>100	>100	>100
**10**	0.37	0.52	1.22	0.41	0.76
**11**	1.77	9.66	16.53	35.68	30.09
**12**	>100	40.08	>100	>100	61.4
**13**	>100	>100	>100	>100	>100
**14**	>100	>100	>100	60.98	>100
**15**	17.71	>100	>100	>100	>100
**16**	69.8	30.02	>100	>100	6.48
**17**	>100	>100	>100	>100	>100
**18**	5.07	>100	91.46	>100	>100
**19**	>100	>100	>100	72.38	47.94
**20**	>100	>100	>100	>100	>100

^a^ key to cell lines employed: A549 (Human Caucasian lung carcinoma); CCD39Lu (Human Caucasian lung fibroblasts); MCF-7 (Human Caucasian breast adenocarcinoma) and MDA-MB-231 (Human Caucasian breast adenocarcinoma).

Most of the compounds did not exert any effects on the non-tumorigenic MCF-12A and CCD39Lu up to 100 μM, being compound **10** the most toxic against both healthy and cancer cells. For the particular case of breast cells, when the compounds were tested in the ER dependent adenocarcinoma MCF-7 cancer cell line, compounds **2**, **4**, **6**, **10**, **11**, **15** and **18** exerted significant activity. However the assay on the estrogen independent MDA-MB-231 cancer cell line revealed a significant degree of toxicity of compounds **10** and **11** and in a lesser extent of compounds **2** and **16**, suggesting that the activity of compounds **6**, **15** and **18** was estrogen dependent. Compound **18** is especially interesting as a potential candidate as therapeutic agent since it is highly toxic and selective towards estrogen dependent MCF-7 cell lines (IC_50_ = 5.07 μM versus more than 100 μM for MDA-MB-231) and almost innocuous for normal breast cells (IC_50_ = 91.46 μM for MCF-12A).

**Fig 2:**
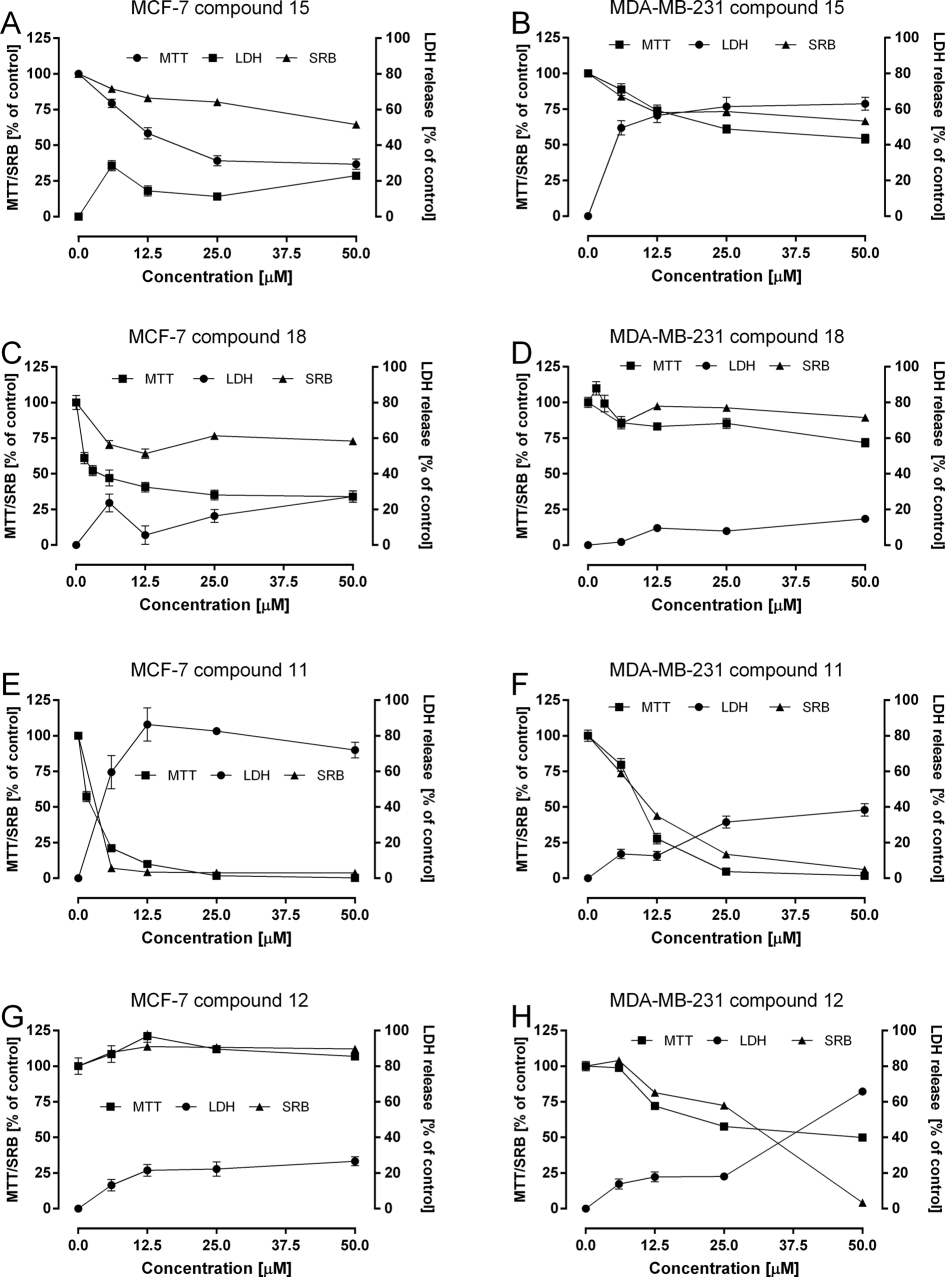
Effect estrogen active compounds 15 and 18 and estrogen inactive compounds 11 and 12 on mitochondrial activity (MTT assay), cell mass (SRB assay) and LDH release in estrogen dependent MCF-7 and estrogen independent MDA-MB-231 cells. Cells were exposed to increasing concentrations of tested compounds for 72 h before performing the assay. Data are reported as % of control and are the means±SEM.

The effects of compounds selected from both groups (selectively active against estrogen dependent cells, compounds **15** and **18**, and compounds without such activity, compounds **11** and **12**) were further assayed in estrogen dependent MCF-7 cells as well as estrogen independent MDA-MB-231 cells. For this purpose, two additional methods, Sulphorodamine B assay **(**SRB) and LDH release assays were employed. While MTT reduction assay measures the dehydrogenases activity in mitochondria, the SRB assay indicates cell density through the measurement of cellular protein content. However, both methods could be considered to measure cell proliferation and viability. On the contrary, the LDH release assay is a direct marker of cell death. This last method evaluate permeabilization of plasma membrane as a result of heavy damage caused by toxic compounds and therefore it is proposed as a method for measuring necrosis and cytotoxicity [39]. In our experiment (**Fig 2**), the proposed compounds selective against estrogen dependent cells **15** and **18** showed a relatively low increase of LDH release (Fig 2A and 2C) in MCF-7 cells. On the contrary, they showed a strong effect on these cells in SRB and MTT assays. It may be stressed that results obtained in MTT were in agreement with values measured in SRB assay suggesting correlation between metabolic activity and cell density. These results may suggest also, that compounds **15** and **18** act as SERMs, since they decrease metabolic activity in MCF-7 cells with much lesser reduction of cell density. This may be in agreement with opinion that the most of SERMs have been identified as antiproliferative compounds. There is no doubt however, that further study are necessary to fully explain SERM like activity of compounds 15 and 18. In MCF-7, the only compound that produces a significant increase in LDH release and cytotoxicity was compound **11** (**Fig 2E**), a compound that is also active in the MDA-MB-231 cell line (**Fig 2F**) and probably is not selective against estrogen dependent cells. Finally, in the MDA-MB-231 cells compound **18** showed non-significant effects on either SRB, MTT or LDH release assays confirming its specificity towards the estrogen receptor bearing MCF-7 cells.

Estrogens and SERMs exert various effects on different types of estrogen dependent cancer cells. Antiestrogens like Tamoxifen suppress breast cancer cell proliferation, while ER agonists may stimulate this process, however it is also very well known that antiestrogens like e.g. Tamoxifen may be involved in the development and proliferation of endometrial estrogen dependent cancer cells [40,41]. Recently a novel ER agonist capable of inducing apoptotic death in various cancer cell was reported. Kaur and coworkers decribed Ospemifene analogues with stronger binding affinities with both ERα and ERβ compared to Ospemifene and Tamoxifen, exerting also cytotoxic effect on estrogen independent MDA-MB-231 cells [42]. On the other hand the novel entrogen receptor agonist inducing apoptotic death in various cancer cell types was described by Kim and coworkers [43].

**Fig 3:**
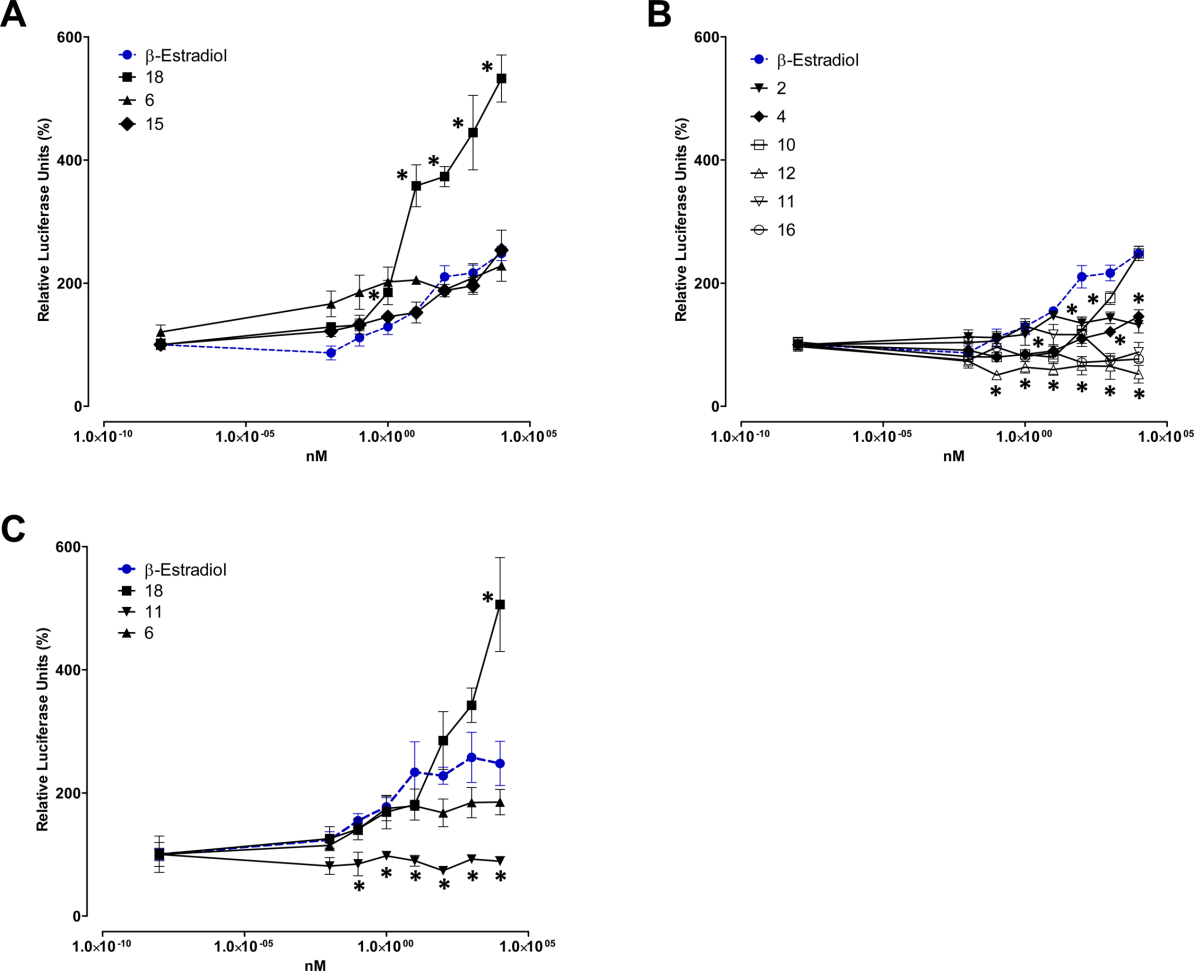
Induction of estrogenic activity of HEK293 cells transfected with ER-α receptor by compounds with selective (A) and no selective (B) toxicity against MCF-7 cells. (C) Estrogenic activity of defined compounds toward MCF-7 cell line. * *p* < 0.05 compared to the control cells treated with β-estradiol.

Since the aim of our research has been the identification of selective estrogen receptor modulators in our library, the effects on the ER dependent transcriptional activity were tested in cultures HEK293 cells transfected with plasmids coding for ERα and an ER dependent reporter system. As a positive control of ER dependent transcription β-estradiol was included. The results presented in **Fig 3** confirmed that compounds **6**, **15** and **18**, with a strong selective activity toward the MCF-7 cells line, are capable to mediate ER dependent transcriptional activity in a similar mode than estradiol. Furthermore, compound **18** showed a higher transcriptional activity at all assayed concentrations compared with estradiol (**Fig 3A**). On the contrary, compounds without selective activity against MCF-7 cells such as compounds **2**, **4**, **11**, **12**, and **16** did not stimulate ER dependent transcriptional activity and only the behaviour of compound **10** was similar to estradiol at the highest concentration assayed (**Fig 3B**).

Next, to confirm the SERM activity of these compounds on the MCF-7 cells, that naturally express both ERs, cells were transfected with the reporter plasmid p3xERRE/ERE-luciferase and the luciferase activity was assayed in the presence of estradiol and the most relevant compounds **(Fig 3C)**. The results obtained in the MCF7 cells mimicked those obtained in the transfected HEK293 cell line. As expected, compound **18** showed a significantly higher transcriptional activity compared to estradiol and although the activity of compound **6** is much lower, it is significantly higher when compared to compound **11**, that is not selective for the estrogen dependent breast cancer cell line and lacks any estrogenic capability.

**Fig 4:**
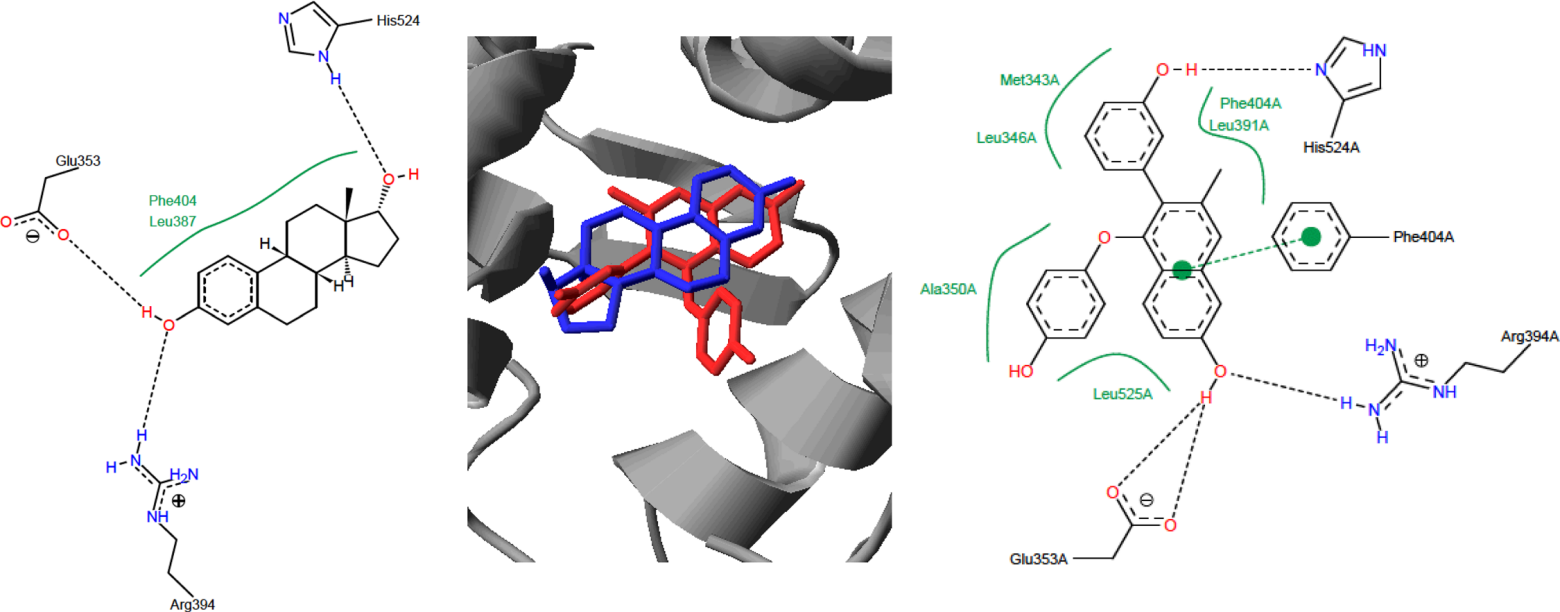
Analysis of the interaction of estradiol (left figure and in blue in the central panel) and GW2368 (right figure and in red in the central panel) from the coordinates computed by docking for estradiol and calculated by X-ray crystallography for GW22368. Dash lines represent hydrogen bonds and green residue labels amino acids with hydrophobic contacts to the ligand.

**Fig 5:**
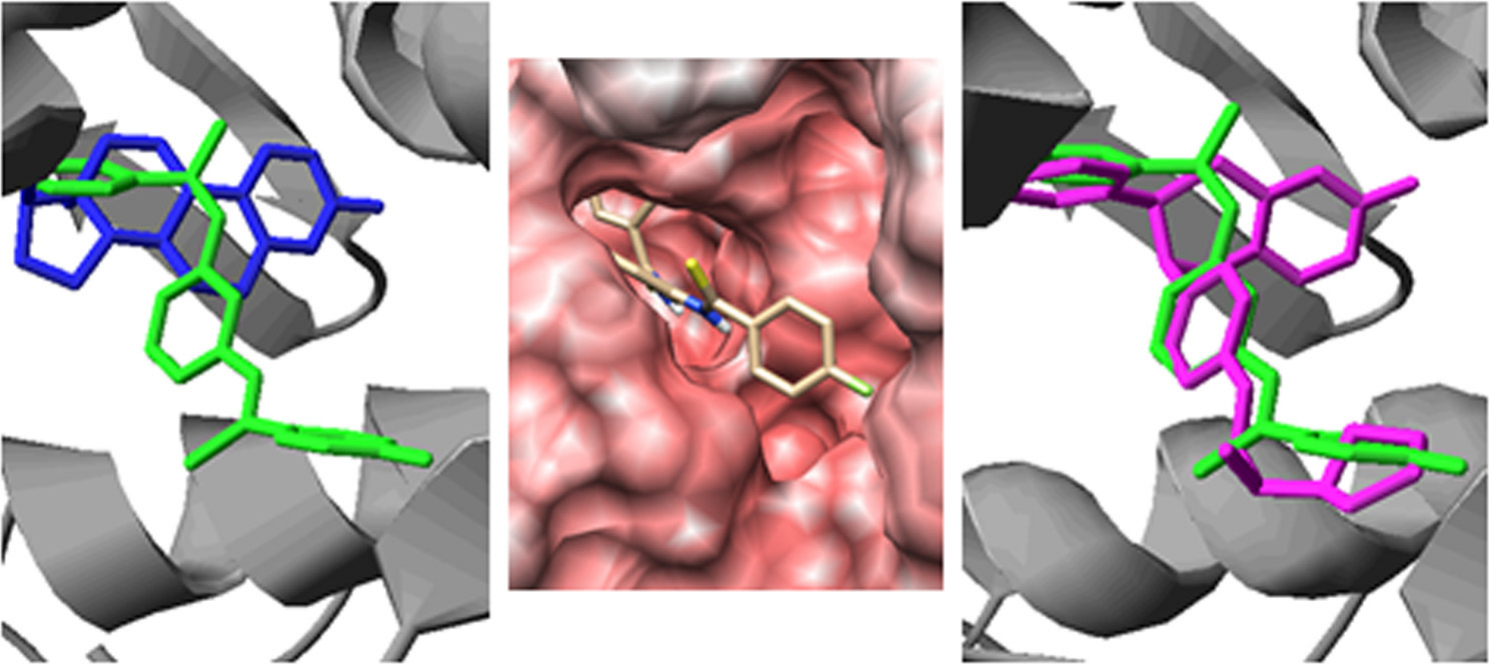
Binding site of 18 (central panel and in green in the others) and comparison with the binding mode of estradiol (Left panel, blue) and ligand AIJ (PDB accession code 1xp9) derived from the dihydrobenzoxathiin scaffold (Right panel, cyan)

To gain additional insight into the high activity of compound **18** as SERM when compared with estradiol, docking studies were carried using the coordinates of the ERα taken from the structure deposited in PDB with the accession code 3dt3 [22]. The computed values of fullFitness and deltaG for the best solutions were -2488.0916 Kcal/mol and -9.096992 Kcal/mol for the docking with estradiol and -2365.7449 Kcal/mol and -8.767751 Kcal/mol with compound **18**. Graphical analysis showed minor differences among the top results of each molecule, supporting the robustness of the calculations. Additionally, the superposition of the structure of the estrogen receptor co-crystallized with the naphthalene analogue GW2368 (PDB accession code 3dt3) [22] onto that obtained from the docking of estradiol yielded a very good fitting of both ligands (**Fig 4**, **central panel**) and further analysis with Poseview [27,44,45] revealed that despite the chemical and structural differences between both ligands, their binding modes share the hydrogen bonding net and some of the hydrophobic contacts. Compound **18** interacts with the receptor in the same cavity as estradiol but the molecule presents an aromatic ring that protrudes and it is involved in extra binding interactions with amino acid W383 (**Fig 5**). In fact, this binding mode resembles that described for dihydrobenzoxantiin estrogen receptor modulators and the pyrrolidine substituent placed at that position has been reported to play an important role [46].

**Fig 6:**
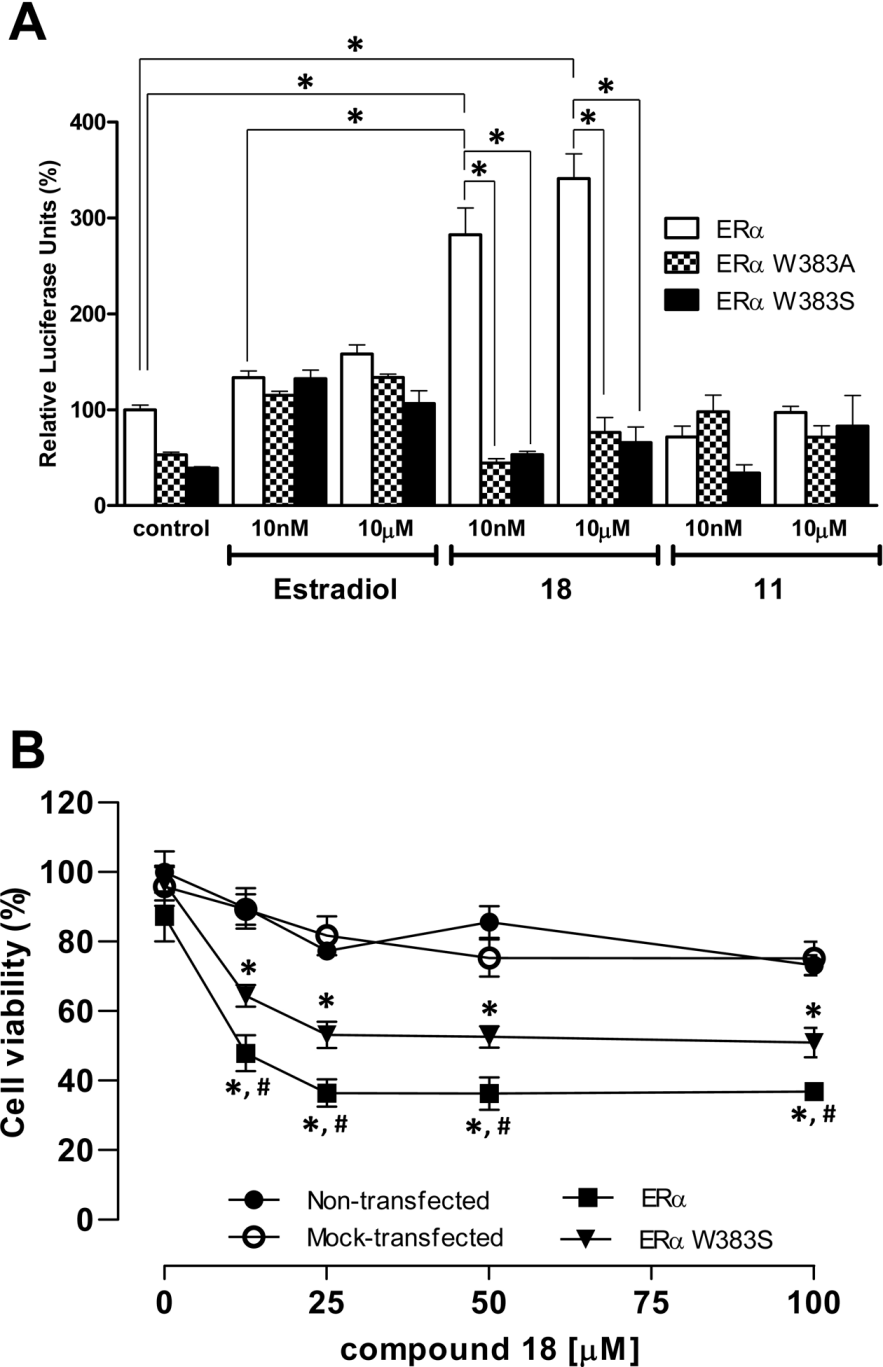
Estrogenic and cytotoxic activity of tested compounds in HEK293 cells transfected with wild type ERα receptor or plasmids bearing mutated versions of ERα. **A**.- HEK293 were transfected with wild type ERα receptor or Erα W383A and W383S plasmids and an ER dependent luciferase reporter gene. Results are expressed as mean ± SEM (n = 8). * *p* < 0.05 was considered statistically significant. **B**.- HEK293 were transfected mock-transfected or transfected with wild type ERα or Erα W383S plasmids. 48 h after transfection, cells were incubated in the presence of increasing concentrations of compounds 18 for 72 h and cell viability was measured by MTT assay. Results are reported as % viability based on the untreated control cells normalized to 100% viable. Results represent means ± SEM (n = 8). * *p* <0.05 versus mock-transfected untransfected cells. # p<0.05 versus ERα W383S transfected cells.

To confirm the relevance of W383 in the binding mode predicted by the docking experiments, plasmids bearing mutated versions of ERα receptor were constructed and HEK293 cells were transfected with either the wild type or the mutated plasmid and the ER dependent transcriptional activity was assayed (**Fig 6A**). The mutations W383A and W383S are predicted to allow an efficient estradiol binding to the ERα receptor as well as preclude the additional interactions in the binding pocket for compound **18**. The results showed that whereas estradiol yields a similar activation of ER dependent transcription in the cells transfected with the wild type or the mutated version of the receptor, compound **18** was less active toward the mutated receptors. This experimental result confirms the docking predictions and highlights the importance of the interaction between W383 and compound **18**. Tryptophan present at position 383 is considered as a conservative point in the hormone binding site and it is also present in other steroids receptors probably due to its longest hydrophobic chain among natural amino acids [47]. Residue W383 interacts with hydrophobic steroid while glutamic acid at position 380 is considered as optimal counterpart potential hydrogen bond acceptor interacting with phenolic group of estradiol which works as hydrogen bond donor [47]. Our biological results support the docking studies and confirm that interaction with W383 is also required for binding **18** with ERα.

Finally, the effects of compound **18** on cell viability were assayed in non-transfected and ERα and ERα W383S transfected HEK293 cells (**Fig 6B**). For that purpose, cells were incubated for 72 h in the presence of increasing concentrations (0–100 μM) of compound **18** and cell viability was determined by MTT assay. While compound **18** showed a moderate cell toxicity in the non-transfected cells, the compound significantly decreased cell viability on the ERα transfected cells. This results point out to the involvement of ERα signaling in the action mode of compound **18.** Furthermore, albeit a decrease in cell viability was observed in the ERα W383S transfected cells, this decrease was significantly smaller compared to the cells transfected with the wild type ERα. This result strength the involvement of ER on the antiproliferative effects of compound **18**.

## Conclusions

In summary, we have demonstrated for the first time the activity of a thiobenzanilide derivative compound **18** as a new, effective and selective estrogen receptor modulator. The studies evaluating its antiproliferative and cytotoxic effects as well as estrogen receptor luciferase assays reveal the selective estrogenic activity of compound **18**. Docking studies predict that it interacts with the ERα receptor in the same cavity as estradiol but with extra binding interactions with position W383 of ERα. This new binding mode was demonstrated by site directed mutagenesis. Although our results show that **18** can act as a very promising SERM, additional experiments are needed to evaluate its potential as a therapeutic agent.
